# The Avon Longitudinal Study of Parents and Children (ALSPAC): an update on the enrolled sample of index children in 2019

**DOI:** 10.12688/wellcomeopenres.15132.1

**Published:** 2019-03-14

**Authors:** Kate Northstone, Melanie Lewcock, Alix Groom, Andy Boyd, John Macleod, Nicholas Timpson, Nicholas Wells

**Affiliations:** 1 Department of Population Health Sciences, Bristol Medical School, University of Bristol, Bristol, BS8 2BN, UK; 2 MRC Integrative Epidemiology Unit, Department of Population Health Sciences, Bristol Medical School, University of Bristol, Bristol, Bs8 2BN, UK

**Keywords:** ALSPAC, Children of the 90s, birth cohort study, cohort profile, enrolment

## Abstract

The Avon Longitudinal Study of Parents and Children (ALSPAC) is a prospective population-based study. Initial recruitment of pregnant women took place in 1990-1992 and the health and development of the index children from these pregnancies and their family members have been followed ever since. The eligible sampling frame was constructed retrospectively using linked recruitment and health service records. Additional offspring that were eligible to enrol in the study have been welcomed through major recruitment drives at the ages of 7 and 18 years; and through opportunistic contacts since the age of 7. This data note provides a status update on the recruitment of the index children since the age of 7 years with a focus on enrolment since the age of 18, which has not been previously described. A total of 913 additional G1 (the cohort of index children) participants have been enrolled in the study since the age of 7 years with 195 of these joining since the age of 18. This additional enrolment provides a baseline sample of 14,901 G1 participants who were alive at 1 year of age.

## Introduction

The Avon Longitudinal Study of Parents and Children (ALSPAC) is a geographically defined, longitudinal birth cohort that recruited pregnant women with an estimated date of delivery between April 1991 and December 1992 ^1, 2^. During the initial recruitment campaign (between 1990 and 1992) women with a total of 14,541 pregnancies were enrolled (some women had more than one eligible pregnancy during this period). The children resulting from these pregnancies and their parents have been followed up ever since, primarily via questionnaire, hands-on measurement at clinical assessment visits (called ‘Focus’ clinics) including the provision and assaying of biological samples and through linkage to routine data. ALSPAC is now a three-generational study, comprising ‘G0’: the cohort of original pregnant women, the biological father and other carers/partners; ‘G1’: the cohort of index children and ‘G2’: the cohort of offspring of the index children. The study website contains details of all the data that is available through a fully searchable data dictionary and variable search tool.

The 2012 G1 cohort profile paper ^1^ describes in detail how the families have been followed up until the age of 18 (G1) and describes how recruitment was extended to include members of families who were eligible to have taken part (using the original eligibility criteria), did not initially enrol but who subsequently wanted to take part. Families were enrolled into the study in *phase I* between 1990 and 1992 during pregnancy and shortly after birth. Further enrolment occurred systematically in *phase II* in 1999 at the age 7 assessment clinic (child mean age: 7.5 years) and then opportunistically in *phase III* from 1999 until 2012 (child mean age: 17.8 years). Since 2012, ALSPAC has conducted an additional systematic recruitment drive and have continued to recruit additional families through opportunistic contacts and through recruitment of the G2 offspring of the index children (in *phase IV* recruitment). This data note provides an update on recruitment *phases II* and *III* and reports specifically on *phase IV* recruitment: that is, all those G1 participants who enrolled in the study from the age of 18 up to and including the clinic assessment held at age 24 (mean age 24.5 years). 

## Methods

Through the Project to Enhance ALSPAC through Record Linkage (PEARL), ALSPAC has retrospectively defined the study’s eligible sampling frame through linking study recruitment records to NHS delivery records and child health records ^1^. This means that the study has a record of the identities (including given and family names; date of birth; NHS ID) of most index children who are eligible to participate. This has allowed ALSPAC to respond to enquiries from both participants and the wider public, for example around eligibility to participate and use of an individual’s data in research projects. For *phase IV* recruitment, since the age of 18 years, there were three ways in which eligible G1 index children (now adults) could enrol into ALSPAC. Firstly, all participants who could be traced were systematically invited to enrol in a postal exercise conducted by PEARL. This was part of their campaign to provide fair processing information to G1 as they reached legal adulthood and as ALSPAC started to systematically link to their health and routine administrative records. Secondly, during recruitment of the G2 participants, the study came into contact with individuals - either as the partner of an enrolled participant or through screening by midwives – who were eligible but had not previously enrolled. Thirdly, eligible participants proactively requesting to enrol are always welcomed and enrolled into the study.

### Study numbers

**Figure 1.**
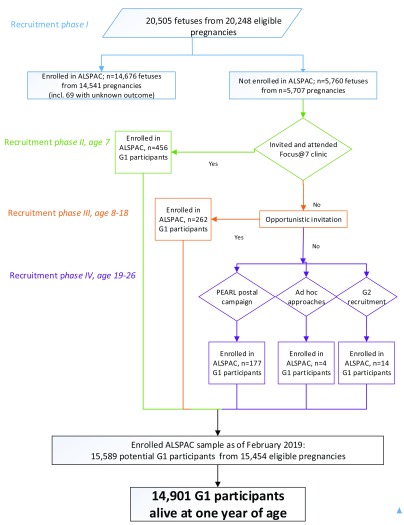
The ALSPAC enrolment campaign flow diagram, illustrating enrolment phases I to IV. (adapted from Boyd *et al*. 2013 ^1^; Oxford University Press, Creative Commons Attribution Non-Commercial License).

A total of 20,248 G0 pregnancies (resulting in 20,505 potential G1 participants) are eligible to take part in the study. Of these pregnancies 116 had an unknown birth outcome. Of the eligible pregnancies, the G0 mothers of 14,676 G1 participants enrolled during the original recruitment campaign (i.e. enrolled in *phase I*). It should be noted that the G0 mothers of 69 pregnancies with unknown outcome have historically always been considered as *Phase I* enrolees, this is appropriate for the G0 cohort profile but not for G1 (since it is not clear how many foetuses were in each of these pregnancies and resulting offspring have not taken part in the study and therefore cannot be adequately quantified). G0 mothers of 456 G1 participants enrolled during the systematic campaign at age 7 (i.e. enrolled in *phase II*). A further 262 G1 participants enrolled through opportunistic contact between the ages of 8 and 18 (i.e. enrolled in *phase III*). This results in a total of 15,394 G1 participants who enrolled by the age of 18 (see Figure 1). Please note, this is a slight increase since the original cohort profile (1) was published.

Since the age of 18, a total of 195 additional G1 participants have enrolled (i.e. enrolled in *phase IV*). Of these, 177 were recruited through systematic postal invitations during 2014. An additional 14 were recruited through the ALSPAC-G2 recruitment campaign: This means that an eligible member of the cohort enrolled after either: i) becoming pregnant, ii) their partner becoming pregnant or iii) they were already a parent. Finally, 4 enrolments resulted from *ad hoc* approaches from eligible participants during this period (see Figure 1). As with previous phases, *phase IV* enrolment included two twin pregnancies where only one twin enrolled. It should be noted that at the time of writing, *phase IV* participants may not have contributed any data to the resource.

A total of 913 (456, 262 and 195 recruited during *Phases II, III* and *IV* respectively) G1 participants have enrolled who were not in the initial study sample (i.e. p *hase I* enrolment). The total sample size for analyses is therefore 15,454 pregnancies, resulting in 15,589 foetuses. Of this total sample of 15,589 potential G1 participants, 14,901 were alive at 1 year of age (it should be noted that self-reported data are not available prior to the time of recruitment for those enrolled in *phase II* or later) and are considered the baseline sample for reporting purposes (14,888 excluding triplets and quadruplets – their data are not generally released for confidentiality purposes). Table 1 summarises the active cases within the study and the potential data available at the time of writing.

**Table 1.** Activity of G1 (the cohort of index children) and illustrative data availability in ALSPAC.

G1 Activity as of *February 2019*	n
Currently active in study *	13,286
Known address	9,856
Can send Qs	8,964
Can send clinic invites	9,306
Linked to Primary health care data	11,810
Linked to Secondary health care data	12,700
At least one DXA ** measure between the ages of 7 and 24	9,144
Genomics data	8,952
Methylation at multiple time points	1,003
G2 parent ^3^	548

DXA - Dual-energy X-ray absorptiometry, G2 - the cohort of offspring of the index children *still alive, not withdrawn, not explicitly opted out **Body composition assessed using Dual-energy X-ray absorptiometry

## Data availability

ALSPAC data access is through a system of managed open access. The steps below highlight how to apply for access to the data included in this data note and all other ALSPAC data:

1. Please read the ALSPAC access policy ( http://www.bristol.ac.uk/media-library/sites/alspac/documents/researchers/data-access/ALSPAC_Access_Policy.pdf) which describes the process of accessing the data and samples in detail, and outlines the costs associated with doing so.
2. You may also find it useful to browse our fully searchable research proposals database ( https://proposals.epi.bristol.ac.uk/?q=proposalSummaries), which lists all research projects that have been approved since April 2011.
3. Please submit your research proposal ( https://proposals.epi.bristol.ac.uk/) for consideration by the ALSPAC Executive Committee. You will receive a response within 10 working days to advise you whether your proposal has been approved.

## Cohort profile data file

The variables described in the cohort profile data file (ALSPAC reference: cp_2b) are provided as a matter of course with all data requests (see Table 2). The denominator used in this file is the 20,505 G1 eligible sample. The cohort profile data is provided in all data extracts, even where participants have formally withdrawn from the study or who are at high risk of disclosure, though it should be noted in these cases their data are supressed.

**Table 2.** Cohort Profile Data File (Version 2b at the time of writing).

Variable name	Variable Label
in_alsp	Enrolled in ALSPAC
in_core	Enrolled as part of original core sample, Phase I
in_phase2	Enrolled as part of phase II, during focus@7
in_phase3	Enrolled as part of phase III, after focus@7 up to age 18
in_phase4	Enrolled as part of phase IV, >=19 & <=24
tripquad	Participant is a triplet or quad
kz011b	Participant was alive at 1 year of age
kz021	Participant sex

If you have any questions about the data or how to access it, please email alspac-data@bristol.ac.uk.

## Consent

Ethical approval for the study was obtained from the ALSPAC Ethics and Law Committee and the Local Research Ethics Committees. Informed consent for the use of data collected via questionnaires and clinics was obtained from participants following the recommendations of the ALSPAC Ethics and Law Committee at the time. Children were invited to give assent where appropriate. Study participants have the right to withdraw their consent for elements of the study or from the study entirely at any time. Full details of the ALSPAC consent procedures are available on the study website ( http://www.bristol.ac.uk/alspac/researchers/research-ethics/).
