# Supplementary material for: The Avon Longitudinal Study of Parents and Children (ALSPAC): an update on the enrolled sample of index children in 2019
Source: Wellcome Open Res. 2019 Mar 14;4:51. [Version 1] doi: 10.12688/wellcomeopenres.15132.1 (PMC6464058; doi:10.12688/wellcomeopenres.15132.1)
Supplement: Peer review history

# Peer review: Reviewer response for version 1

**DOI:** 10.21956/wellcomeopenres.16511.r35067

**Published:** 2019-04-10

**Authors:** Aisling Murray

**Affiliations:** 1 Economic and Social Research Institute (ESRI), Dublin, Ireland

**Overview**

 This data note provides a brief summary of later additions to the ALSPAC G1 cohort (i.e. the original children). As I understand from the note, these were individuals who would have been eligible to participate but did not actually enrol in the study until a later date. Summary details are given on the number of individuals, the broad stage at which they joined and how they were recruited – particularly given the most recent joiners between the ages of 18 and 24 years.

**Opinion**

 The main points of the note are generally clearly described and benefit from the inclusion of the diagram in Figure 1, which breaks down the numbers joining and at what stage. There are two exceptions: (a) in Table 1, it is not clear whether the n for ‘known address’ and ‘linked to Primary health care data’ etc. are subsets of the 13,286 described as ‘currently active’ in row 1 or the full 14,901 participants in the final cell of Figure 1; (b) in the abstract, the term “additional offspring” implies later children who were born to the same mother could enrol but in the main note text that does not appear to be the case.

 I have no particular criticisms of the note, and it should be a useful resource for those using (or considering using) the ALSPAC data. There are some additional points that would be ‘nice to know’ however. Perhaps the authors could consider either including them either (briefly) in the note or adding a reference to direct the reader to the relevant information elsewhere? They are as follows:

- Do we know why the late joiners did not originally participate? Had their mother initially refused or were they not identified as eligible at the start? The note suggests the latter might have been the case for some.
- A brief reminder of the eligibility criteria at the start would be useful. I assume, for example, that only people initially born in the Avon area be able to join the study later and not those who immigrated there at a later date, but I am not certain from reading the note in isolation.
- How similar is the demographic profile of the late-joiners to the originally enrolled sample (e.g. gender, ethnicity, social class, health status)? And have the study weights been adjusted to account for the characteristics of the new joiners?
- The note makes brief mention of cohort ‘G0’, the mothers of the original children known as ‘G1’. If a data user were considering using G0 data, it would be good to know if (a) any of the mothers of the late joiners also entered the study in their own right and (b) if they stay in the study even if their G1 children leave.

I have read this submission. I believe that I have an appropriate level of expertise to confirm that it is of an acceptable scientific standard.

# Peer review: Reviewer response for version 1

**DOI:** 10.21956/wellcomeopenres.16511.r35066

**Published:** 2019-04-03

**Authors:** Hazel Inskip, Sarah Crozier

**Affiliations:** 1 MRC Lifecourse Epidemiology Unit, University of Southampton, Southampton, UK; 2 MRC Lifecourse Epidemiology Unit, University of Southampton, Southampton, UK; 3 NIHR Southampton Biomedical Research Centre, University Hospital Southampton NHS Foundation Trust and University of Southampton, Southampton, UK

This is a useful paper for anyone using the ALSPAC resource, setting out how the additional participants have been included at different stages. The innovative methods have been reported in detail and we anticipate that the availability of this Data Note will enhance understanding. We do however have some suggestions that might aid clarity:

 We found the use of the term ‘index’ somewhat confusing. ‘Index’ to us rather implies the first children recruited. This paper describes the additional recruitment of children after the original (index) children had been recruited. Maybe stick to the term G1, where necessary, after defining it?

 Also we felt that there is a confusion in the title in that it implies that the additional children were all enrolled in 2019. Could it be reworded to say “a 2019 update on the enrolled sample of index children”?

 The abstract could do with a little clarification. The words ‘additional offspring’ is slightly confusing as it sounds as though it’s additional children born to the original mothers. As eligibility hasn’t been defined at this point, it’s not easy to follow what this means. Maybe the authors could define eligibility in the abstract and then make it clear that those children who would have been eligible, but who were missed originally, have later been included in the study.

 Also in the abstract it would be helpful if the word ‘additional’ could be inserted before ‘recruitment’ in the sentence: “This data note provides a status update on the recruitment of the index children since the age of 7 years with a focus on enrolment since the age of 18, which has not been previously described”.

 When you say that the original eligibility criteria were applied retrospectively, did you extract the expected date of delivery for all them to check this, or was it based on date of birth?

 It would be helpful if reference to Figure 1 was moved to near the top of the Study numbers section as it is useful to look at it while reading the subsequent paragraph.

 In the first paragraph on study numbers, there are these two sentences: “G0 mothers of 456 G1 participants enrolled during the systematic campaign at age 7 (i.e. enrolled in phase II). A further 262 G1 participants enrolled through opportunistic contact between the ages of 8 and 18 (i.e. enrolled in phase III).” At age 7, presumably the G1 participants were enrolled in addition to their mothers? In the enrolment between ages 8 and 18 were no G0 mothers enrolled or was it just the children? Also were any G0 participants enrolled in phase IV?

 In the sentence “As with previous phases, phase IV enrolment included two twin pregnancies where only one twin enrolled.” it is not clear whether both twins were ever included in any phase. The flowchart implies not, but it would be helpful if this could be clarified.

 We found the numbers in the top three boxes of the flowchart somewhat confusing. The 14,676 + 5,760 fetuses do not sum to 20,505 in the top box, whereas the 14,541+5,707 pregnancies do sum to the 20,248 in the top box. The 69 are included in the pregnancies but not in the fetuses in the left-most box, and this isn’t clear.

 At the end of the second paragraph of the Study numbers, it might be helpful to add something like “but will be included in subsequent data collection waves”.

 The following sentence was initially confusing: “The cohort profile data is provided in all data extracts, even where participants have formally withdrawn from the study or who are at high risk of disclosure, though it should be noted in these cases their data are supressed.” It seems odd to say that the profile data are provided for everyone but then to say that the data are suppressed for particular participants. It might help to modify the end of the sentence to say “… in these cases other data are suppressed”. (Please note that “suppressed” has been typed incorrectly).

 Minor comments:

 There seems to be an extraneous “(G1)” in the second line of the second paragraph of the introduction.

 At the end of the first paragraph of Study numbers section, reference number 1 should superscripted.

 The sentence in the last paragraph of the introduction that starts: “Since 2012, ALSPAC has conducted an additional systematic recruitment drive and have continued to recruit additional families through….” makes ALSPAC singular and plural. We suggest that ‘have’ should become ‘has’.

 Similarly there is inconsistent use of data as plural and data as singular in the last sentence of the first paragraph of the Cohort profile data file section.

 In Table 1, it’s pretty obvious that Qs stands for questionnaires but there is room to spell it out in full.

We have read this submission. We believe that we have an appropriate level of expertise to confirm that it is of an acceptable scientific standard.
